# Appetitive Pavlovian conditioning in two inclusive kindergarten classrooms

**DOI:** 10.1002/jeab.70107

**Published:** 2026-05-18

**Authors:** Richard A. Colombo, Saba Mahmoudi, Waylon B. Wright, Ya‐Hsuan Huang, Gregory J. Madden

**Affiliations:** ^1^ Department of Special Education University of Washington Seattle Washington USA; ^2^ Department of Psychology Utah State University Logan Utah USA

**Keywords:** appetitive Pavlovian conditioning, conditioned reinforcement, human, inclusion, special education

## Abstract

The present study evaluated the efficacy of a classwide appetitive Pavlovian conditioning protocol implemented in two inclusive kindergarten classrooms. Each classroom enrolled similar distributions of typically developing children and children who were either eligible for special education services or diagnosed with autism spectrum disorder. Two Pavlovian conditioning trials (CS+ ➔ US) and two unpaired trials (CS‐ ➔ no US) per day were superimposed on normal classroom activities at random times. Conditioned responses (i.e., positive emotional responding and attention directed to the CS) were recorded during the CS+ and CS‐ events. In both classrooms—one in which the teacher verbally specified the Pavlovian contingency and one in which they did not—the CS+ evoked significantly more of both conditioned responses in typically developing children. Children with developmental disabilities showed significantly more CS+‐evoked attention, but the effect size was smaller than among typically developing children. The CS+ did not evoke significantly more positive emotional responding in the children with developmental disabilities unless the teacher verbally specified the CS+ ➔ US contingency. Such procedural refinements may be important if a therapeutic appetitive CS is to influence adaptive operant behavior via, for example, conditioned reinforcement or Pavlovian‐to‐instrumental transfer in children with developmental disabilities.

Pavlovian conditioning occurs when an individual learns a temporal relation between a conditioned stimulus (CS) and an unconditioned stimulus (US). Prior to this learning, the CS is a neutral stimulus (NS), but after Pavlovian conditioning, the CS evokes one or more conditioned responses. Pavlovian learning is adaptive because it allows organisms to prepare for phylogenetically important US events before they occur (Krause & Domjan, 2017). If the US is food, salivating when the CS is presented (e.g., the smell of barbeque) prepares us for digestion. In the wild, the smell of an invasive‐species predator (CS) often precedes an attack (US), and organisms that learn this CS ➔ US temporal sequence are more likely to survive because the odor alone (CS) evokes changes in heart rate, blood pressure, pupil size, attention, etc. (Lennartz & Weinberger, 1992), all of which prepare the organism to avoid attack.

Pavlovian learning also influences operant behavior. A CS that reliably precedes an aversive US will evoke fear responses—like freezing—that interfere with and decrease ongoing operant behavior (Estes & Skinner, 1941). Conversely, a CS signaling an imminent appetitive US can increase operant responding through Pavlovian‐to‐instrumental transfer (PIT; Cartoni et al., 2016). More specifically, a PIT effect occurs when response‐independent presentations of the CS increase some dimension of operant responding, relative to CS‐absent intervals. Finally, operant responding is increased when an appetitive CS is provided contingent on an operant response—a conditioned reinforcement effect (Williams, 1994).

Despite the importance of Pavlovian learning to adaptive behavior in general, and operant responding in particular, arranging Pavlovian CS ➔ US contingencies is not a common procedure used in applied behavior analysis. Although applied behavior analysts frequently use token reinforcers and praise, the reinforcing function of these stimuli are rarely established through Pavlovian procedures; instead, they are typically established through verbal explanations (token reinforcers) or rely on preexperiment histories (praise). Where Pavlovian conditioning procedures have been arranged to promote adaptive behavior or to establish a new conditioned‐reinforcer function, the results are often inconsistent (see Argueta et al., 2024, for a review).

Madden et al. (2023) suggested that some of this inconsistency may be due to training procedures that overlook one or more of the six principles of effective Pavlovian conditioning listed in Table 1. The most ignored of these is Principle 3: arrange a large *C/T* ratio. The *C/T* ratio is relevant to the timing of US and CS events in a Pavlovian conditioning protocol. The components of the *C/T* ratio are the average inter‐US interval (*C*) and the average time to the US during the CS (*T*). When the *C/T* ratio is large (e.g., 1,200 s/12 s = 100), the onset of the CS signals a large reduction in the delay to the US (i.e., a 100‐fold delay reduction in this example). Larger *C/T* ratios facilitate Pavlovian learning, improve the evocative function of the CS, and—consistent with Fantino's delay reduction theory (Fantino et al., 1993; Shahan & Cunningham, 2015)—a CS established with a large *C/T* ratio is a more effective conditioned reinforcer than one established with a small *C/T* ratio (see Madden et al., 2023, for a review).

**TABLE 1:** Six principles of Pavlovian learning and conditioned reinforcement.

Principle	Description
1: Choose a salient and novel NS.	Salient and/or novel NS events are more likely to be observed, and this is critical if the individual is to learn the temporal relation between NS and US.
2: Arrange an effective US/backup reinforcer.	Pavlovian learning is facilitated if the US is a phylogenetically important event or in the case of appetitive Pavlovian learning, an effective reinforcer.
3: Large *C*/*T* ratios are better than small ones.	Pavlovian learning is facilitated when the NS signals a large reduction in the time to the next US delivery. That reduction is quantified by the *C/T* ratio (see text for details).
4: The NS must uniquely inform the individual that the US is coming.	If an established CS already signals that the US will arrive sooner than normal, the NS is unlikely to acquire a CS function.
5: Fewer trials per session, faster per‐trial learning.	Arranging more trials per session slows Pavlovian learning on a per‐trial basis.
6: A reliable NS–US contingency facilitates acquisition; an intermittent contingency increases resistance to Pavlovian extinction	The NS will acquire a CS function faster if the NS reliably precedes the US. However, Pavlovian extinction (presenting the CS without the US) will happen faster if the CS–US contingency remains in place. Following acquisition, greater resistance to Pavlovian extinction can be achieved by presenting the US probabilistically after the CS.

*Note*: NS = neutral stimulus; US = unconditioned stimulus; *C* = the inter‐US or interreinforcer interval; *T =* the average time to the US during the CS.

A practical limitation of arranging large *C/T* ratios in applied settings is the opportunity cost of dedicating long periods to *C* (e.g., a 20‐min interval between US events), time that could be spent in therapeutic or academic activities (Taylor et al., 2025). Although Pavlovian conditioning is facilitated by arranging fewer trials per session (Principle 5), even arranging a session with three such CS ➔ US trials would take an hour. A potential solution to this practical limitation is to superimpose Pavlovian conditioning trials on ongoing activities. Specifically, arranging a handful of appetitive CS ➔ US trials per school or clinic day ensures a large *C/T* ratio. This may be a challenge in a noisy setting where the salience of the CS may be low, but if it was successful, it would be an efficient Pavlovian conditioning procedure because all students or clients would learn the CS ➔ US contingency simultaneously. Thereafter, the appetitive CS might be used as a conditioned reinforcer or a response‐facilitating stimulus (PIT) or to increase self‐control choice (e.g., Garland & Madden, 2025).

Another potential benefit to improving Pavlovian conditioning procedures in a natural setting like a classroom is the ability to learn more about and to assist students with autism spectrum disorder (ASD) and other developmental delays. The few studies comparing Pavlovian learning in children with and without ASD have reported mixed outcomes. South et al. (2011, 2012) reported no differences in acquisition of the CS function, although South et al. (2012) found that the ASD sample showed impaired Pavlovian reversal learning. More consistent impairment of Pavlovian learning was reported by Oristaglio et al. (2013) and Powell et al. (2016). All these studies were conducted in laboratory settings using an aversive US (i.e., loud noise or puff of air to the eye) and physiological dependent measures (e.g., galvanic skin conductance) during Pavlovian training. Publicly observable behavioral differences involving appetitive Pavlovian conditioning in nonlaboratory settings have yet to be evaluated.

The present experiment sought to evaluate whether superimposing Pavlovian contingencies on normal activities in two inclusive kindergarten classrooms could establish a new appetitive CS function. Consistent with the principles in Table 1, a salient CS+ was presented for 18 s before a preferred US (fun activities), with two CS+ ➔ US trials per day, each separated by more than 2 hr, on average. Thus, the onset of CS+ signaled a large reduction in the delay to the next US event. To ensure that the CS+ was the only stimulus that signaled the US, teachers were blinded to the timing of these events. This ensured that they did not inadvertently signal that a trial was about to begin, which—in accordance with Principle 4—could decrease training efficacy. Finally, to evaluate control by the CS+, an equally salient CS‐ was presented twice per day with the same intertrial interval. The US never occurred within 5 mins of a CS‐ event. Two conditioned responses were measured: positive emotional responses and attention directed to the CS+ or CS‐. We hypothesized that the CS+ would evoke more positive emotional responses and more attention than the CS‐. A secondary purpose of the present experiment was to evaluate potential Pavlovian learning differences between the typically developing children and children who were either eligible for special education services or were diagnosed with ASD.

## METHOD

### Participants

The experiment was completed sequentially in two inclusive kindergarten classrooms on the campus of the University of Washington; all participants were between the ages of 5 and 6 years. The first classroom enrolled 15 students, eight were typically developing (three male), three had special education eligibilities (SPED; two male), and four were independently diagnosed with ASD (all male). The second classroom also enrolled 15 students, seven were typically developing (three male), five had SPED (all male), and three were independently diagnosed with ASD (all male). The sample size was determined by the number of students available in the two classrooms, which represents a convenience sample. The study protocol was reviewed and approved by the institutional review board at the University of Washington (STUDY00018926; MOD00020086).

### Setting and materials

Each classroom was staffed by one teacher and three classroom assistants and equipped with typical kindergarten furnishings including small tables, a rug with seating squares, a whiteboard, colorful decorations, a small library, a play area, and a sink. The researchers provided the teacher in each classroom with an Alexa Dot and an Alexa Glow (hereafter referred to as the Dot and Glow; Amazon, Seattle, WA), which were simultaneously used to present either the CS+ or CS‐. The Dot was placed on a flat surface approximately 1.22 m high at the front of the classroom. The Glow, which presented visual stimuli, was placed on a 15.24‐cm box next to the Dot. The devices were programmed to play music (Dot) and present colors (Glow) using the Amazon Alexa app.

Researchers supplied the teachers with items to be presented as the US. These items, which the teachers predicted the children would enjoy, included silk dance scarves, balloons, bubbles, a bubble machine, and pop‐up books. Each classroom was equipped with two hardwired, dome cameras (M5526‐E, Axis, Lund, Sweden) installed in the ceiling and equipped with video‐ and audio‐recording capabilities. Video Audio Learning Tool (VALT; Sussex, WI) software was used for recording and storing the videos securely within the system.

### Procedures

#### Discriminated Pavlovian conditioning

The experiment was completed first in Classroom 1 and then in Classroom 2. During each day of the discriminated Pavlovian conditioning phase, two CS+ ➔ US trials and two CS‐ ➔ no‐US trials occurred at times the teacher was blinded to. Each trial type was presented once in the morning and once in the afternoon during 1‐hr windows during which the teacher said all children were likely to be in the classroom. During CS+ trials, the Glow illuminated blue while the Dot played *Blue Train* by John Coltrane for the initial 18 s of the song at 80% volume, which was deemed by the researchers and teachers to be loud enough to be heard above the competing sounds of the classroom. Following the CS+ presentation, the teacher transitioned to the US activity within 5 s. If the US activity required distributing materials, prior to CS+ onset the teacher kept the item in an inconspicuous but accessible location and made it visible to all participants as soon as the CS+ was turned off. The teacher provided all children with 3 to 5 min of access to the US activities, which were rotated through to reduce habituation and improve the value of the US (Egel, 1981; Hanratty & Hanley, 2021). US activities included a scarf party (tossing scarves into the air while music plays), a balloon “keep‐up” game (while standing on rug squares, trying to keep a balloon aloft), a bubble party (children chase and pop bubbles created by a bubble machine), a dance party (the teacher turned on music and turned off the lights and let the children dance), a reading from a pop‐up book, or access to age‐appropriate YouTube videos. No food items were used as the US event. During CS‐ trials, the Glow illuminated with green light for 18 s while the Dot played the first 18 s of *Blue Skies* by Art Tatum at the same volume. No programmed events followed CS‐ events. Classroom 1 completed 18 CS+ and CS‐ trials, whereas Classroom 2 completed 20 of each of these trial types.

During the first 3 days of discriminated Pavlovian conditioning in Classroom 1 (six trials), the teacher broke from the intended protocol by teaching the children to discriminate between CS+ and CS‐ trials. That is, the teacher directed student attention to the audio‐visual display of the Dot and Glow, described what was happening (e.g., “Look, its blue”), and said either “When it's blue, we get to do something fun,” or “When it's green, we go back to work.” Rather than terminate the experiment in this classroom, we continued, reasoning that other teachers may do the same if this protocol were deployed elsewhere. In Classroom 2, the teacher and aides were explicitly instructed to provide no information about the CS+ or CS‐ events. If students asked about these stimuli, they provided a neutral response (e.g., “I'm not sure. What do you think?”) and provided no additional feedback.

#### Direct observation methods and operational definitions

The dome cameras and microphones in each classroom were used to record the 18 s prior to and the 18 s of the CS+ and CS‐ events. Graduate‐level research assistants independently observed these recordings and used partial‐interval recording to quantify the classroomwide frequency of two responses made before and during the stimulus events: *positive emotional responses* and *attention directed to the CS+ or CS‐* (operational definitions below). The behavior of each child present was recorded. The 18 s before and during each stimulus event was broken into six contiguous 3‐s intervals. For each child, if responding occurred at any time during an interval, it was recorded as a positive interval; intervals without a response were recorded as a negative interval. If a student was out of the camera frame for more than two observation intervals, they were excluded from data collection for that trial. Separate elevation scores were calculated for each response by subtracting the proportion of positive intervals occurring in the 18 s before the stimulus presentation from the proportion of positive intervals during the 18‐s CS+ or CS‐ event. For example, if a participant made positive emotional responses in four of the six observation intervals during the CS+ and in one of the six intervals before that stimulus event, their elevation score was 0.667–0.167 = 0.5 for that trial. If a participant was outside of the camera frame for up to two of the 3‐s observation intervals (e.g., during the pre‐CS interval), the same number of randomly selected observation intervals on the other side of the stimulus onset (e.g., during the CS) were excluded to keep the number the same within each trial. If a participant was outside of the camera frame for more than two 3‐s observation intervals, that participant's data on that trial were excluded from the final analysis. Additionally, sometimes it was possible to see only a subset of a participant's responses (e.g., cheering and jumping but not smiling because the child's back was to the camera). When this occurred, data for this dependent measure were gathered only for the observable responses, always maintaining an equal number of observation intervals on either side of the stimulus event.

Positive emotional responses included smiling, jumping/dancing, or cheering. Smiling was operationally defined as a facial expression change characterized by an open‐mouthed grin with visible teeth or, if the mouth was closed, noticeable upward movement in the corners of the mouth. Jumping/dancing was defined as any repeated up‐and‐down or side‐to‐side swaying movement of the body—whether the participant was standing, seated, or kneeling. Cheering was defined as any louder than normal vocalization that included positive statements like “Whoo!” or “Yea, blue!” If any students made negative vocalization at a louder‐than‐normal level (e.g., “Not green again!”), it was not counted as cheering.

The other recorded response was attention directed toward the Dot and Glow. Human laboratory Pavlovian conditioning studies often report an attentional bias toward an appetitive CS, as measured by eye tracking (Heck et al., 2025). Thus, attention was operationally defined as (a) orienting the head or body toward or pointing at the CS+ or CS‐ for at least 1 s or (b) physically moving toward these stimuli.

#### Interobserver agreement, procedural fidelity, and social validity

Interval‐by‐interval interobserver agreement (IOA) was calculated for 31% of the trials in Classroom 1 and 30% in Classroom 2, half CS+ and half CS‐. All participants who were present during a trial were included in IOA observations. Agreement was defined as both observers recording either the occurrence or nonoccurrence of a response within each of the recorded intervals. The IOA was calculated by dividing the agreements by the total number of agreements plus disagreements and converting the ratio to a percentage. Mean IOA across these trials was 98% in Classroom 1 (range: 83% to 100%) and 97.4% in Classroom 2 (range: 83% to 100%).

Procedural fidelity was assessed on a randomly selected 33% of the trials in Classroom 1 and 30% of the trials in Classroom 2. For these assessments, we evaluated whether the teacher (a) allowed the CS+ or CS‐ to be played for the full 18 s, (b) refrained from providing instructions (Classroom 2 only), and (c) delivered the US within 5 s of turning off the CS+ and withholding the US on CS‐ trials. Except for refraining from providing instructions, the Classroom 1 teacher implemented trial components with 100% accuracy, whereas the Classroom 2 teacher implemented all components with an average of 97.3% accuracy.

Both teachers completed a social validity survey at the conclusion of the study. The three‐item survey asked teachers to use a 5‐point Likert scale (1 = *strongly disagree* to 5 = *strongly agree*) to indicate whether the protocol was (1) feasible in their classroom, (2) enjoyed by their students, and (3) easy for the teacher to understand.

#### Statistical analyses

Separate elevation scores were calculated for positive emotional responses and attention directed toward the Glow and Dot. For each elevation score, the proportion of the six observation intervals containing a conditioned response in the 18 s before the stimulus (CS+ or CS‐) was subtracted from the proportion of intervals with a conditioned response during the 18‐s stimulus (values could range from −1 to 1). Nearly all individual participants had missing data because they were either absent or out of the camera frame. The data were multilevel by design, with repeated presentation of the two stimuli nested within participants, nested within different instruction conditions. Linear mixed‐effects models were used to account for the multilevel nature of the data and to accommodate missing data (Hox et al., 2017).

Primary analyses evaluated whether elevation scores differed between CS+ and CS‐ trials (main effect of stimulus). Models also included diagnostic categories (typically developing, SPED, or ASD) and instruction conditions as fixed effects, along with theoretically motivated interactions among stimulus, diagnosis, and instruction. Participant was included as a random effect to account for within‐subject dependence.

For the *attention to the CS* dependent measure, the model that included main effects of stimulus, diagnosis, and instruction, along with the Stimulus × Diagnosis interaction, provided a superior fit compared with a full model that also included interactions involving instruction. The additional interaction terms did not meaningfully improve model fit or explained variance, as evaluated using AIC, BIC, and likelihood ratio tests, and were therefore excluded to retain a best model.

For the *positive emotional response* dependent measure, the best fitting model retained interactions between stimulus and diagnosis and between stimulus and instruction but excluded the three‐way interaction. This model provided a better fit (AIC, BIC, etc.) and was used for the final analysis. Effects of trial numbers were explored in initial analyses but were not used in the final models due to minimal contribution to overall model fit.

Post hoc pairwise comparisons were conducted using estimated marginal means to compare diagnostic groups within each stimulus and instruction condition as well as stimulus conditions within each diagnosis and instruction group. To control multiplicity, *p* values were adjusted using the false discovery rate procedure. The full data set and analysis files are available on Open Science Framework at: https://osf.io/ec8wq/?view_only=e6c0eba528f04cbfb0a512dc1608639e.

An exploratory post hoc analysis evaluated whether stereotypic behavior occurring among SPED and ASD children may have inhibited Pavlovian learning. More specifically, if these children were engaged in stereotypic behavior at times when the CS+ or CS‐ was presented, this may have inhibited learning the temporal relation between these stimuli and the US. Stereotypy was operationalized as (a) flapping hands in front of their face for > 2 s, (b) repeated physical movements inappropriate to the current activity (e.g., jumping while peers were seated for rug time), (c) waving objects in front of their eyes, or (d) lining up objects. For each SPED and ASD child, five CS+ and five CS‐ trials were randomly selected and scored as 1 (stereotypy present during the stimulus) or 0 (no stereotypy). Because of the small sample size and the comparable effects in SPED and ASD participants, data were merged across diagnoses. Two separate Mann–Whitney *U* tests (one for CS+ and the other for CS‐ trials) evaluated whether participants with stereotypy in one or more trials had lower mean attention elevation scores than participants who never engaged in stereotypy during that trial type.

## RESULTS

**FIGURE 1:**
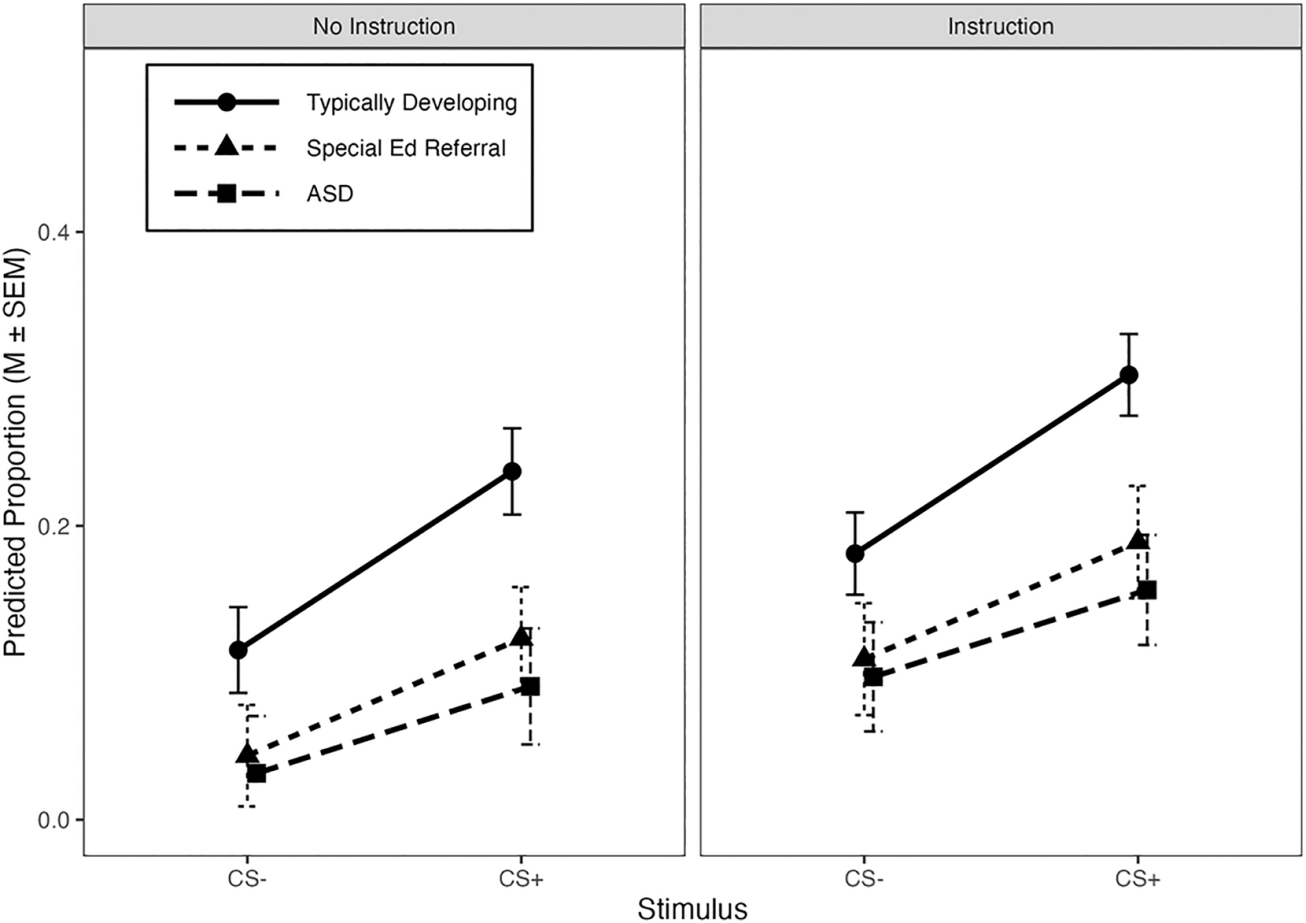
Model‐predicted elevation scores (±SEM) for attention directed toward the CS+ and CS‐ (*x*‐axis). Data are separated by group (symbols) and whether instructions were provided (panels). The *y*‐axis range extends to 0.5. ASD = autism spectrum disorder.

Figure 1 shows model‐predicted attention directed to the CS+ and CS‐ events (elevation scores); data are separated by instructions (panels) and diagnostic category (symbols). Indicative of a Pavlovian conditioning effect, there was a significant main effect of stimulus (*p* < .001), with more participant attention directed to CS+ than CS‐ events. Although there was a nominally significant main effect of instruction, *t*_(25.96)_ = 2.09, *p* = .05, there were no significant interactions involving instructions. Thus, instructions increased attention directed to both CS+ *and* CS‐ events, and this increase was unaffected by diagnosis. The Stimulus × Diagnosis interaction was significant (*p* = .04), reflecting that sensitivity to stimulus type (CS+ vs. CS‐) depended on diagnosis. Said another way, typically developing children showed larger increases in CS+ over CS‐ attention scores relative to SPED and ASD children. Post hoc comparisons revealed significantly higher CS+ than CS‐ attention elevation scores in typically developing, *t*_(708.43)_ = 8.87, *p* < .001; *d* = 0.92, 95% CI [0.70, 1.14]; SPED, *t*_(708.53)_ = 3.85, *p* < .001; *d* = 0.57, 95% CI [0.26, 0.87]; and ASD, *t*_(707.29)_ = 2.62, *p* < .01; *d* = 0.41, 95% CI [0.09, 0.72], children.

**FIGURE 2:**
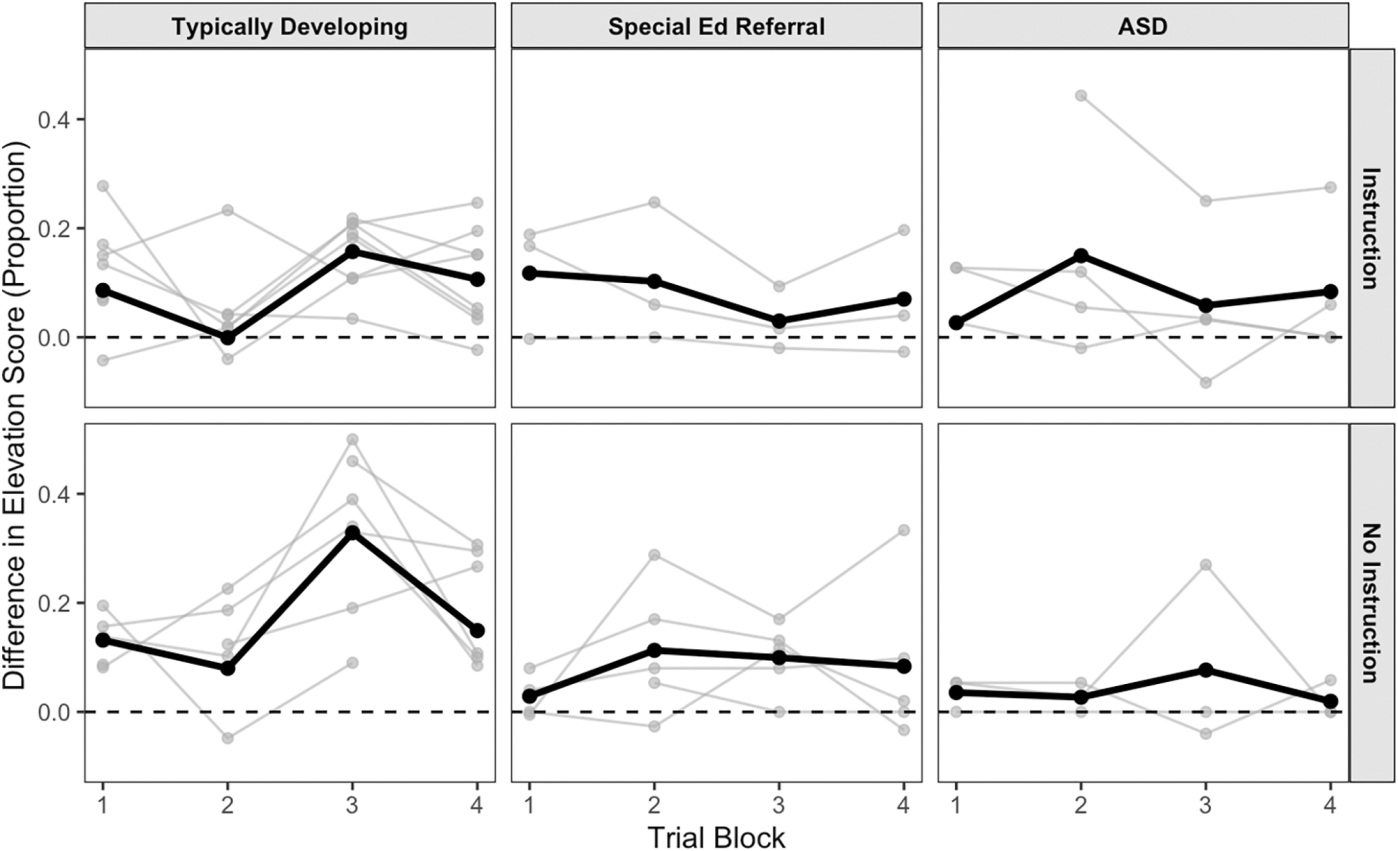
Trial‐block level differences in attention elevation scores across CS+ and CS‐ for individual children (gray lines) in the three diagnostic categories (rows) and instructional conditions (columns). Solid lines provide group means. ASD = autism spectrum disorder.

Figure 2 shows differences in attention elevation scores (i.e., CS+ scores minus CS‐ scores) across the course of the training phase. Data are organized into four 5‐trial blocks (i.e., the sum of CS+ proportional elevation scores in 5 trials minus the sum of CS‐ proportional elevation scores in the same trials). Because children in one classroom completed 18 CS+ and 18 CS‐ trials, their first trial block contained only three (instead of five) CS+ and CS‐ trials. Light gray lines show individual participant data, and group means are given by black lines. Data are separated by diagnostic categories (rows) and instructional conditions (columns). Among typically developing children, difference scores were generally positive, indicating that attention was more often evoked by the CS+ than the CS‐ across instructional conditions. The same was true of SPED and ASD children, although the differences were smaller among uninstructed ASD children.

**FIGURE 3:**
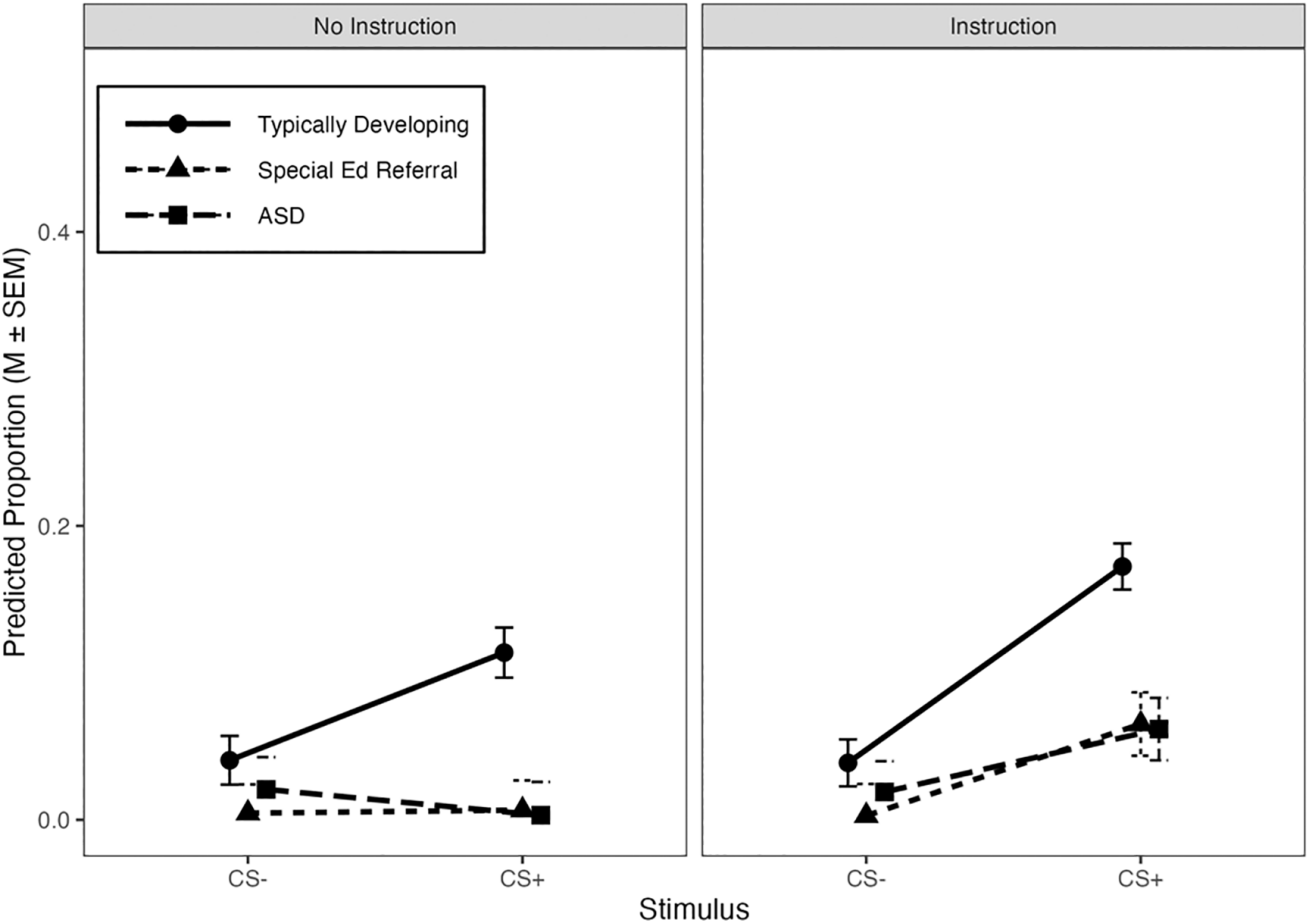
Model‐predicted elevation scores for positive emotional responses evoked by the CS+ and CS‐. Data are separated by group (symbols) and whether instructions were provided (panels). The *y*‐axis range extends to 0.5. ASD = autism spectrum disorder.

Figure 3 shows model‐predicted elevation scores for the second dependent measure: positive emotional responses evoked by CS+ and CS‐ events. Although there is a main effect of stimulus (*p* < .001), this effect was influenced by instructions (Stimulus × Instructions interaction, *p* < .001) and diagnosis (Stimulus × Diagnosis interaction, *p* < .001); the three‐way interaction was not significant. The significant Stimulus × Diagnosis interaction reveals that the CS+ evoked more positive emotional responses than the CS‐ in typically developing children with (*d* = 1.34, 95% CI [1.08, 1.60]) or without instructions (*d* = 0.78, 95% CI [0.51, 1.05]), but this was not consistently observed among SPED or ASD children. For these groups, the CS+ evoked more positive emotional responses than the CS‐ if they received instructions—SPED: *t*_(712.67)_ = 3.65, *p* < .001; *d* = 0.61, 95% CI [0.27, 0.96]; ASD: *t*_(710.29)_ = 2.50, *p* < .05; *d* = 0.42, 95% CI [0.08, 0.75]—but not when instructions were omitted (*p*s > .401).

**FIGURE 4:**
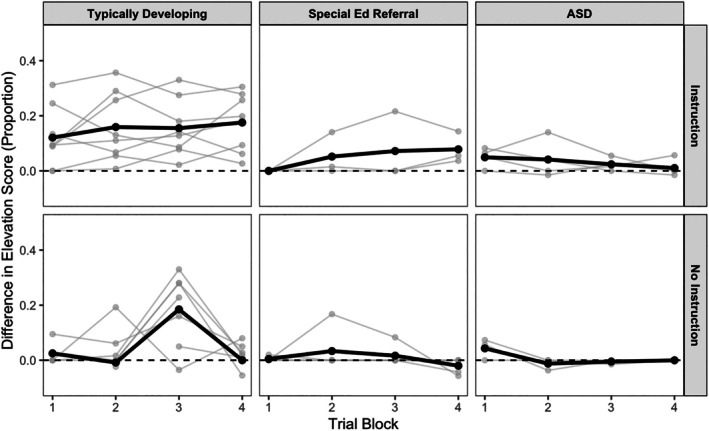
Trial‐block level differences in positive emotional response elevation scores across CS+ and CS‐ for individual children (gray lines) in the three diagnostic categories (rows) and instructional conditions (columns). Solid lines provide group means. ASD = autism spectrum disorder.

Figure 4 shows trial‐block‐level differences in positive emotional response elevation scores across CS+ and CS‐ for all participants across the three diagnostic categories and instructional conditions. As was true of attention, among typically developing children, the CS+ more often evoked positive emotional responding than the CS‐ (positive differences). For SPED and ASD children, positive emotional responses were rarely evoked by either the CS+ or CS‐, so the difference scores mostly hovered around zero.

**FIGURE 5:**
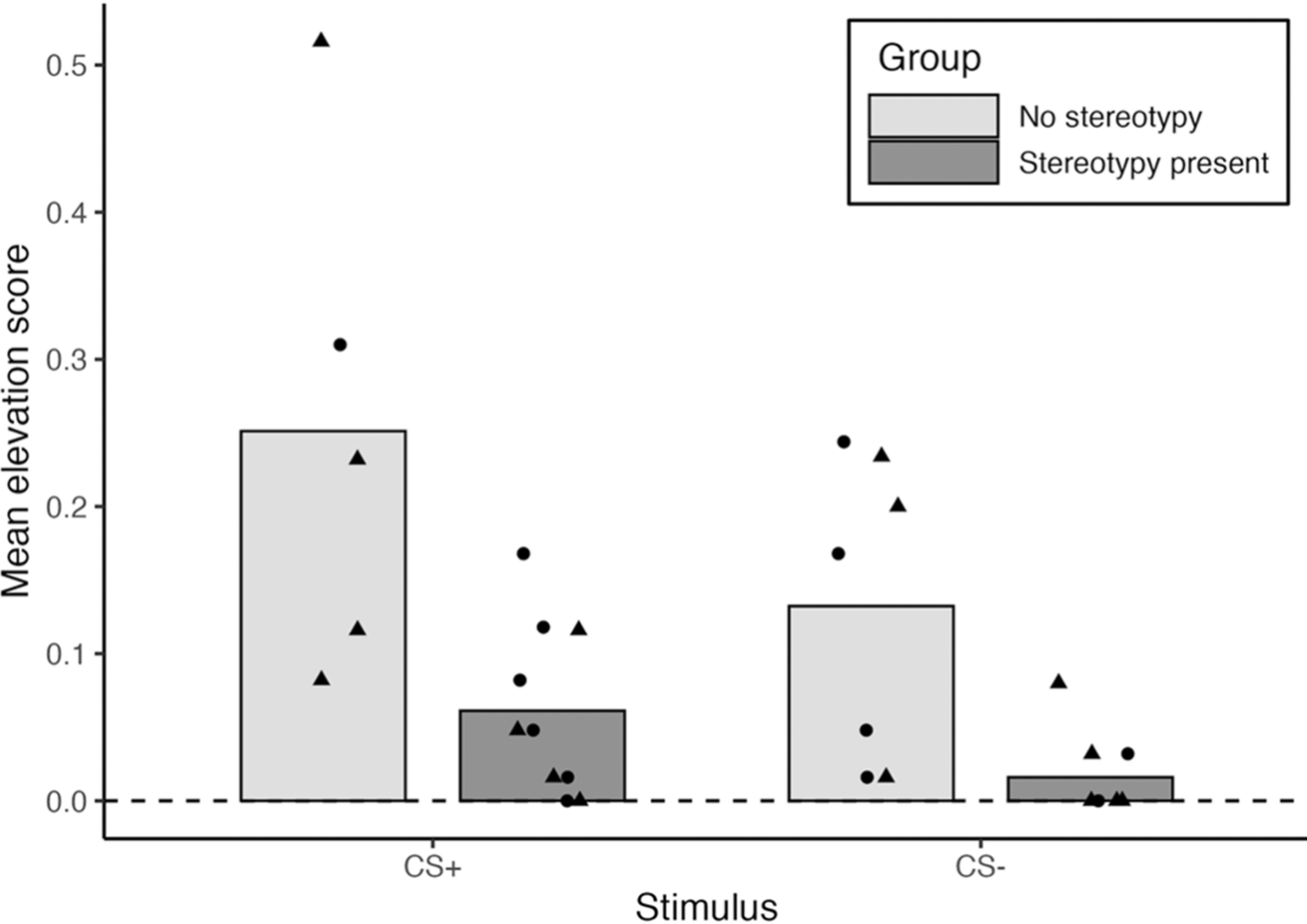
Individual participant and mean elevation scores for attention to the CS+ and CS‐. Data are separated by SPED (triangles) and ASD (circles) children who engaged in no stereotypy or some stereotypy during the CS+ or CS‐ trials.

Figure 5 shows the results of the exploratory analysis evaluating whether stereotypic behavior may have interfered with Pavlovian learning, as quantified by attention elevation scores. Regardless of trial type (CS+ or CS‐), SPED and ASD children who engaged in any stereotypy attended to the Pavlovian stimulus significantly less than SPED and ASD children who never engaged in stereotypy: CS+ (*U* = 29, *p* = .023) and CS‐ (*U* = 21, *p* = .016).

Teacher‐completed social validity survey outcomes depended on classroom. The teacher who explained the Pavlovian contingency to the children (Classroom 1) somewhat agreed that the protocol was feasible and enjoyable (4 out of 5) and strongly agreed that it was easy to understand (5 out of 5). The teacher who was asked not to explain the Pavlovian contingency somewhat disagreed that the protocol was feasible or enjoyable (2 out of 5) and was neutral about how easy it was to understand (3 out of 5).

## DISCUSSION

This study demonstrates, for the first time, the feasibility of establishing a new appetitive CS function using a Pavlovian conditioning protocol overlayed on the daily routines of two inclusive kindergarten classrooms. Conditioned responding was measured across all students, revealing differences among diagnostic groups (typically developing, SPED, and ASD). Nearly all typically developing children acquired the appetitive CS function (evoking attention and positive emotional responding), whereas children in the SPED and ASD groups showed comparatively less responding. This was reflected in lower elevation scores for attention directed toward the CS and inconsistent evidence of CS‐evoked positive emotional responding.

The demonstration that appetitive Pavlovian conditioning can be successfully implemented with high implementation fidelity on a classwide basis while imposing nominal opportunity costs (i.e., no need to schedule long‐duration Pavlovian training sessions) suggests this procedure might be practically used to establish a CS function. Once established, this appetitive CS might then be used to increase adaptive operant responding through either PIT (i.e., presenting an antecedent CS to increase operant responding) or conditioned reinforcement (i.e., presenting a response‐contingent CS to increase the future probability of operant responding; Madden & Falligant, in press). As summarized by Argueta et al. (2019), Pavlovian protocols for establishing these CSs have a mixed track record during the acquisition phase in the applied behavior‐analytic literature. This may occur because one or more of the Table 1 principles of effective Pavlovian learning are not adhered to. The present training protocol was designed to adhere to those principles (e.g., large *C/T* ratio, few trials per day, CS reliably signaled a reduction in the delay to the US), and the training outcomes were robust among the typically developing children. The potential therapeutic use of an appetitive CS requires such consistent initial training outcomes before their utility may be properly assessed when integrated into a behavior‐analytic intervention. Having said that, applying Pavlovian principles is a challenge for those of us with limited training in Pavlovian conditioning. Those wishing to explore the utility of Pavlovian conditioning in applied settings would do well to collaborate with experts in that form of learning, particularly those who understand Pavlovian learning from an information‐theoretic orientation (see Cunningham, 2026).

Returning to the present study, training outcomes were less robust among the children with a SPED referral or an ASD diagnosis. This finding is consistent with laboratory studies reporting poorer Pavlovian learning outcomes among individuals with ASD (Oristaglio et al., 2013; Powell et al., 2016). In the present study, our exploratory post hoc analysis revealed that SPED and ASD children who engaged in stereotypy during some training trials had poorer learning outcomes. If stereotyped behavior prevented a child from observing the CS+ prior to the fun activity, this would inhibit their ability to learn the CS+ ➔ US relation (Principle 1). Stereotyped behavior occurring during a CS+ may also have been incompatible with conditioned responses that would have been evoked by the CS+/CS‐ (e.g., attention to the CS+) had the stimulus been observed. Similarly, language deficits among SPED and ASD children may have hindered the ability of the CS+/CS‐ to evoke positive vocalizations. Finally, it is also possible that the fun activity was less appetitive for SPED and ASD children who engaged in more stereotypy (Principle 2). Future research should systematically evaluate and ameliorate these possibilities—for example, by pretesting the salience of the CS+, evaluating the reinforcing efficacy of the “fun activities,” and exploring other conditioned responses that are more appropriate to SPED and ASD populations.

In their study of Pavlovian learning in children, Powell et al. (2016) reported that “explicit awareness”—the ability to describe the Pavlovian CS–US contingency—was correlated with better conditioning outcomes in children with ASD but not in typically developing children. That finding accords with our results showing that the CS+ did not evoke positive emotional responses in ASD children unless the teacher provided a verbal description of the Pavlovian contingency. Future research might evaluate the effects of supplementing the present Pavlovian conditioning protocol with a prompting‐and‐fading procedure designed to help language‐capable ASD participants to describe the CS–US contingency.

Across diagnostic groups, the CS+ evoked more positive emotional responses after the teacher provided verbal instructions about the Pavlovian contingency. Although this could be due to “explicit awareness,” it may also have occurred because the instructions implicitly signaled that positive emotional responding was acceptable and would not be punished in the academic setting. For children who were not instructed, positive emotional responses were relatively muted. That attention directed toward the CS+ was unaffected by instructions is consistent with this hypothesis, as the children were given greater freedom in the classroom to explore different learning activities during much of the school day.

A limitation of the present study concerns use of music as the CS+ and CS‐. In both classrooms, either stimulus onset occasioned dancing, which was a programmed activity in the classroom. Because dancing was a behavior in the operational definition of a positive emotional response, this may account for some responding in the presence of the CS‐. Future studies may benefit from using simpler auditory stimuli (e.g., different tones) to reduce this potential confound.

Teachers differed in their assessment of the social validity of the Pavlovian training protocol. The teacher providing more positive ratings (4 to 5 out of 5) was more experienced (10+ years in the classroom) and had participated in prior research conducted by other investigators. The teacher providing lower ratings (2 to 3 out of 5) had worked at the school for only 1 year and had not participated in prior classroom studies. Another possible reason for the higher social validity ratings in one classroom is that the CS+ evoked more consistent positive emotional responding in that classroom. This was the same teacher who provided instructions about the CS+. Thus, it is possible that the higher social validity ratings were influenced by happier children during the CS+ and the lower ratings were controlled by less child enthusiasm and more confusion about the function of the CS+ and CS‐ stimuli.

Given these outcomes, it is worth asking whether teachers, therapists, or caregivers should instruct language‐able children about the Pavlovian CS–US contingency. Although typically developing children learned the CS+ function with or without instructions, if social validity is reliably lower when instructions are omitted, dissemination of the protocol will be more difficult. Among the SPED and ASD participants, instructions facilitated positive emotional displays during the CS+, which may improve social validity and conditioned reinforcer efficacy. That said, “rule‐governed” Pavlovian learning may be different from Pavlovian‐contingency‐shaped learning. For example, it is common in applied behavior analysis to verbally describe the Pavlovian contingency between a conditioned reinforcer (CS) and the back‐up reinforcer (US). Although this rule facilitates acquisition of contingency learning, the CS may lose its conditioned reinforcing function if the Table 1 principles of effective Pavlovian learning are not adhered to (e.g., if the *C/T* ratio is low and, therefore, the CS signals little reduction in the delay to the back‐up reinforcer). In the absence of more empirical data, it may be prudent to arrange Pavlovian contingencies that reliably produce uninstructed acquisition. Doing so increases the probability that the CS will retain its conditioned reinforcing efficacy over time.

Finally, Madden et al. (2023) discussed how appetitive Pavlovian conditioning might be leveraged to create a CS that increases self‐control choice. Given the present study's success in establishing an appetitive CS in typically developing children using a classwide protocol with nominal opportunity costs, future studies should investigate whether the self‐control‐enhancing outcomes observed in rodents (Garland & Madden, 2025; Mahmoudi & Madden, 2024) can be systematically replicated in human children. If, for example, children are more tolerant of delays (e.g., less inappropriate behavior while waiting for a delayed reward) when given an appetitive CS during the delay, then teachers, therapists, and caregivers may be excited to begin a Pavlovian training protocol so that they can obtain a tool for reducing impulsivity and promoting patience. Thus, future work must evaluate strategies to improve the acquisition of the appetitive CS function for children with SPED and ASD.

## AUTHOR CONTRIBUTIONS

**Richard A. Colombo:** conceptualization, investigation, funding acquisition, writing – original draft, methodology, validation, visualization, writing – review and editing, project administration, data curation, supervision, formal analysis, resources. **Saba Mahmoudi:** conceptualization, writing – original draft, methodology, validation, visualization, formal analysis, data curation. **Waylon B. Wright:** writing – original draft, data curation, resources. **Ya‐Hsuan Huang:** writing – original draft, resources, data curation. **Gregory J. Madden:** conceptualization, writing – review and editing, visualization, validation, methodology, formal analysis, data curation, supervision, resources.

## CONFLICT OF INTEREST STATEMENT

The authors have no conflict of interest to declare.

## ETHICS APPROVAL

This study was reviewed by the University of Washington's Institutional Review Board (STUDY00018926; MOD00020086). This study received exempt status, which did not require individual participant consent. Instead, a passive consent procedure was used: An informational flyer was sent home, and guardians could choose to opt out. This process was approved by the University of Washington's Institutional Review Board and the research site.

## Data Availability

The full data set and analysis files are available on Open Science Framework at https://osf.io/ec8wq/?view_only=e6c0eba528f04cbfb0a512dc1608639e.
